# Current perspectives for the treatment of chronic myeloid leukemia

**DOI:** 10.3906/sag-1810-81

**Published:** 2019-02-11

**Authors:** Elifcan ALADAĞ, İbrahim Celalettin HAZNEDAROĞLU

**Affiliations:** 1 Department of Hematology, Faculty of Medicine, Hacettepe University, Ankara Turkey

**Keywords:** Chronic, myeloid, leukemia, tyrosine, kinase, inhibitors

## Abstract

With an annual incidence of 1–2 in a million, Ph*(+) chronic myeloid leukemia (CML) is a clonal hematopoietic stem cell disease that makes myeloid neoplastic cells breed out of control. This BCR-ABL(+) myeloproliferative disease makes up about 15%–20% of all leukemia cases in adults. CML is seen more in males than females, with a rate of three to two. However, it does not show differences in prevalence in terms of age. CML consists of three clinical phases. The first one is the chronic phase, defined by rising white blood cell levels and also by myeloid proliferation and bone marrow maturation. While this phase does not exhibit complications, in diagnosis, it comprises most of the patients. The second phase is the accelerated phase, which the disease progresses to if it is not treated or does not respond to treatment. This usually takes about 3 years. The third phase is the blastic phase. The chronic phase can still progress to the next two phases within the first 2 years, with a rate of 10%. In the following years, the possibility increases by 15%–20% each year. Tyrosine kinase inhibitors (TKIs) are revolutionary drugs for the management of disease course in CML. The aim of this review is to assess current approaches to CML patients’ follow-up and treatment with TKIs. A literature search on CML and TKIs was made in PubMed, Web of Science, and Scopus with particular focus on randomized clinical trials, recommendations, guidelines, and expert opinions. In managing CML, various treatment methods have been utilized for many decades. Prior to the development of TKIs, interferon alpha was the primary tool, which was then complemented by allogeneic hematopoietic stem cell transplantation (HSCT). HSCT was successful in slowing the disease down in the long term and curing up to 50% of patients. Then the coming of the imatinib era opened up different treatment perspectives. For the patients resistant or intolerant to imatinib, second- and third-generation TKIs are successfully used in distinct CML disease states. The survival benefits of TKIs including imatinib, nilotinib, dasatinib, bosutinib, and ponatinib for CML patients are outstanding. TKI-related adverse events could impact the clinical course, especially in long-term drug administrations. The current aim for CML disease management in the TKI era is to provide age- and sex-matched normal life duration to CML patients.

## 1. Introduction

Defined by the spreading of myeloid neoplastic cells in the circulating blood and in the bone marrow, chronic myeloid leukemia (CML) is a malignant clonal hematological disease. The existence of the Philadelphia (Ph) chromosome causes the fusion transcript BCR-ABL to form. The pathological activity of BCR-ABL leads to myeloid neoplasia as well as genomic instability [1]. Medications that inhibit BCR-ABL formation, called tyrosine kinase inhibitors (TKIs), are helpful in restoring normal hematopoiesis and providing hematological, cytogenetic, and molecular alleviation. These medications can also facilitate this with accurate monitoring of patients [2]. If the inhibitors fail in limiting the BCR-ABL, the disease potentially progresses on to the accelerated phase or the blastic phase. This progression leads to death in 3 years [3]. As is the case with various chronic benign diseases, like diabetes and hypertension, rationally administrating these inhibitors can be related to normal age- and sex-expected life duration [4]. In this review, we intend to summarize the existing TKI treatment methods in CML along with future treatment aspects.

## 2. The definition, classification, and scoring of CML 

CML was defined as the discovery of t(9;22) (q34.1;q11.2) by traditional cytogenetics along with the discovery of BCR-ABL1 by molecular genetic practices by the World Health Organization in its revision of myeloid neoplasm classification in 2016. This classification also included complete karyotype and bone marrow morphologic findings to verify the myeloid disease. The revision describes the accelerated phase of CML depending on the morphologic, hematological, and cytogenetic parameters. These parameters are completed by genetic evolution and TKI resistances. They describe the blastic phase based on the occurrence of at least 20% blasts in the circulating blood or bone marrow or on extramedullary accumulation being observed in blastic cells [5]. There are other cytogenetic anomalies (Iso(17q), additional Ph, +19, -7, 3q26, +8) that can cause complications in CML [6].

In composing long-term treatment plans for TKI-naïve patients, prognostic evaluation of CML is key [4]. Prior to the use of inhibitors, Sokal CML scoring was a widely used system. It is based on the age, spleen, blasts, and platelets of the patient. Similarly, another system of the same era, Euro-Hasford, takes the same into account in patients, with the addition of eosinophils and basophils. The single system used in the era of TKIs is EUTOS, which is based only on spleen and basophils. However, the long-term survival evaluation of EUTOS adds age and blasts [4]. 

## 3. Frontline treatment of CML

Using TKIs orally is quite common for CML patients in the present period. Imatinib mesylate at 400 mg/day is common as the frontline treatment, whereas some extraordinary cases may require dasatinib at 100 mg/day or nilotinib at 600 mg/day [7–10]. There are inhibitors available that are more powerful than these, although they should be reserved for patients with risky Sokal scores or complex karyotypic anomalies. Determining the risk score is key for newly diagnosed patients. For frontline treatment, the following should be taken into consideration: patients’ comorbidities, preferences, individual characteristics, drug toxicity profiles, and physician experiences [8,10–12].

### 3.1. Imatinib mesylate as frontline treatment

From previous studies on the ideal dose of imatinib to be used for CML patients, the frontline treatment is recommended as imatinib mesylate at 400 mg/day. Table 1 shows the comparison of inhibitor doses in those studies.

**Table 1:** Main studies comparing different imatinib doses used in CML treatment.

Clinical study	Major critical conclusion	CCyR 12 months with imatinib	MMR 12 months with imatinib
IRIS	Imatinib is superior to IFN+Ara-C treatment with regard to hematological, cytogenetic, and molecular responses (imatinib is the ‘standard of care’ in CML)	68%	38%
TOPS	High-dose imatinib may obtain faster but not more responses	66% (w/400 mg) 70% (w/800 mg)	40% (w/400 mg) 46% (w/800 mg)
ELN/GIMEMA STUDY (only high-Sokal CML)	High-dose imatinib could not increase responses in high-risk CML	58% (w/400 mg) 64% (w/800 mg)	36% (w/400 mg) 43% (w/800 mg)
GERMAN CML STUDY IV	Imatinib dose optimization may obtain earlier and more molecular responses (addition of non-PEG IFN is not useful)	49% (w/400 mg) 63% (w/800 mg)	34% (w/400 mg) 46% (w/800 mg)
FRENCH SPIRIT	PEG-IFN and imatinib combination may obtain earlier, deeper, and more molecular responses (increasing the dose of imatinib is not useful)	58%	38%
ENESTnd	Nilotinib is superior to imatinib with regard to cytogenetic and molecular responses	65%	22%
DASISION	Dasatinib is superior to imatinib with regard to cytogenetic and molecular responses	72%	28%
TIDEL	CML treatment optimization with imatinib dosage, plasma level monitorization, and convenient shift to nilotinib is possible with better responses	88%	47%
BELA	Bosutinib may obtain more molecular (but not cytogenetic) responses than imatinib	68%	27%

Kantarjian et al. suggested 800 mg/day imatinib to be more efficient than 600 mg/day for frontline treatment. While the high-dose imatinib group of that study exhibited complete cytogenetic response (CCyR) by 90% and major molecular response (MMR) by 63%, side effects such as hematotoxicity, hepatotoxicity, and skin rash were also seen in the same group [13]. In a study that applied 800 mg/day imatinib mesylate as the frontline treatment in patients with risky Sokal scores, the rates observed at 12 months and 24 months were as follows: CCyR at 88% and 91% and MMR at 56% and 73%, respectively. The rates of side effects were even higher than those found by Kantarjian et al., this time including myalgia, nausea/vomiting, and diarrhea [14].

Hughes et al. compared 600 mg/day imatinib mesylate with 800 mg/day and found the response rates for the 800 mg/day group at 12 months and 24 months to be as follows: CCyR at 88% and 90% and MMR at 47% and 73%, respectively [15].

Deininger et al. did the same with an 800 mg/day group compared to a group receiving the standard dose (400 mg/day). Their findings showed CCyR by 85% in the 800 mg/day group at 12 months and by 67% in the 400 mg/day group, while at 12 months MMR was seen by 53% and 35% in the 800 mg/day and 400 mg/day groups, respectively [16].

Other than studies that support higher doses of imatinib mesylate to be more efficient, there are also studies that support higher doses of imatinib to be more effective, such as the American TKI Optimization and Selectivity (TOPS) study, the Central European Imatinib Standard, or the ELN vs. High Dose Imatinib Trial study.

In the TOPS study, where 400 mg/day or 800 mg/day imatinib mesylate was given randomly to patients as frontline treatment, 12-month response rates were observed as follows: CCyR at 70% and 66% and MMR at 46% and 44% in the 800 mg/day group and the 400 mg/day group, respectively. These differences were not significant, along with the progression of disease. However, hematological and nonhematological side effects were seen more in the 800 mg/day group [17].

 Baccarani et al. studied the same dose groups as the TOPS study in their ELN study, although they only studied patients with risky Sokal scores. As was the case with the TOPS study, they did not have findings supporting higher doses of imatinib. The 12-month response rates were observed as follows: CCyR at 64% and 58% and MMR at 40% and 33% in the 800 mg/day group and the 400 mg/day group, respectively [18].

 The Central European Leukemia Group conducted their study by applying 400 mg/day imatinib after 800 mg/day application to CML patients as the frontline treatment, and CCyR rates were significantly worse whereas MMR rates were seen to be higher at 6 months in the group receiving the higher dose [19].

Organic cation transporter 1 (OCT1) is defined as an influx transporter facilitating imatinib transportation to CML cells. Hughes et al. reported evident differences in MMR rates and survival rates between patients who were given 600 mg/day imatinib with high OCT1 activity and low OCT1 activity [20]. The study suggests that OCT1 activity acts as a determinative factor for MMR and progression-free survival rates in patients where imatinib is used as the frontline treatment. It can also be helpful in detecting patients requiring higher doses of imatinib in frontline treatment [20].

### 3.2. Second-generation TKIs in frontline treatment

Second-generation TKIs, dasatinib and nilotinib, show stronger response and tolerability compared to imatinib, making them more suggestible as frontline treatment for patients with risky Sokal scores. There have been two studies on the comparison between these second-generation TKIs and imatinib in patients with CML.

In the ENESTnd study, with groups receiving 400–300 mg nilotinib twice a day and a 400 mg/day imatinib mesylate group, statistically significant differences were observed. The nilotinib groups exhibited higher MMR and CCyR rates at 24 months. The response rates were as follows: CCyR and MMR were respectively at 87% and 71% in the 300 mg nilotinib group, 85% and 67% in the 400 mg nilotinib group, and 77% and 44% in the imatinib group [10]. Similarly, the DASISION study was conducted with a 100 mg/day dasatinib group and a 400 mg/day imatinib group. The 24-month response rates were as follows: CCyR at 86% and 82% and MMR at 64% and 46% in the dasatinib group and the imatinib group, respectively. As these two studies have shown, these second-generation TKIs show better response rates as frontline treatment methods in patients with CML. 

The NiloPeg study was conducted with patients with CML in the chronic phase, where nilotinib at 300 mg twice a day was given to patients along with Peg-IFN for 1–2 years, and they were only given nilotinib past the 2-year mark. The combination period’s CCyR rates at 6 and 12 months were 71% and 100%, respectively, while the MMR rates at 12, 24, 36, and 48 months were 76%, 78%, 83%, and 73%, respectively [21]. A similar study, PETALS, compared a 600 mg/day nilotinib group with a 600 mg/day nilotinib + Peg-IFN group in de novo Philadelphia-positive patients with CML in the chronic phase. The collection of findings in these studies showed that nilotinib combined with Peg-IFN is an applicable and efficient method for better, earlier, and deeper MMR [21,22].

### 3.3. Which TKI is the best method as the frontline treatment of CML patients?

There are a number of treatment methods that can be considered for CML patients thanks to the new studies conducted.

Method 1: As the frontline treatment, 400 mg of imatinib is safe to be prescribed to any patient, which should be followed by a second-generation TKI in the case of resistance or intolerance. So far, there is no determinative difference in the choice of a second-generation TKI, so any one of the medications with a positive pharmacoeconomic characteristic is safe to be prescribed.

Method 2: Based on the Sokal scores of patients, 400 mg of imatinib or a second-generation TKI can be prescribed for low or high risk scores, respectively. 

Method 3: Any one of the second generation TKIs or imatinib may be prescribed as the frontline treatment.

Imatinib treatment can have some side effects, of which the most widely observed one is swelling in periorbital and lower extremities. It is observed with high frequency in patients older than 65 years and in patients who are given high doses. While the side effects can mostly be alleviated with diuretics, prescribing one of the second-generation TKIs may still prove safer as the frontline treatment [23,24]. Another side effect of imatinib treatment is the occurrence of bone marrow suppression, mostly appearing during the first few months of therapy. In addition, hypothyroidism is seen, at 2 weeks on average, in patients who receive thyroid hormone treatment before taking imatinib, where levothyroxine doses may be doubled [25].

As dasatinib can lead to fluid retention along with pleural effusion and pericardial effusion, in patients with pleural effusion or with a high risk of pleural effusion occurrence, prescribing another inhibitor would be better. Dasatinib may also lead to pulmonary arterial hypertension, in the treatment of which stopping dasatinib induction can help improve hemodynamic parameters, as vasodilator agents do not exhibit clinical advantages [26,27]. For the side effect of bone marrow suppression, which occurs due to dasatinib induction, particularly in advanced cases of CML, adjusting the doses can help reverse the side effect. Another side effect observed due to dasatinib induction is platelet dysfunction [10,28].

**Figure 1:**
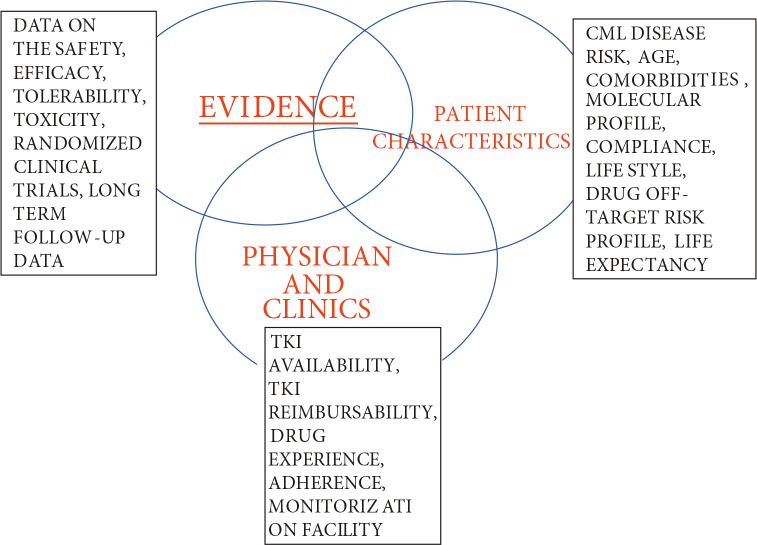
Clinical decision making for any tyrosine kinase inhibitor (TKI) drug in chronic myeloid leukemia (CML). Clinical decision should be reached based on the optimization of the best available evidence, individual patient/disease characteristics, and clinical experience.

The other second-generation TKI, nilotinib, may deteriorate glucose levels in blood, which is not a case seen with the two other inhibitors. Another side effect of nilotinib is QT interval prolongation, which is not seen frequently but is lethal, so it should be monitored at the beginning of the treatment and throughout the whole process [10,29]. Nilotinib is also stated to be related to cardiovascular disorders, which were observed to increase in occurrence with induction by nilotinib (8% for nilotinib 300 mg, 13% for nilotinib 400 mg, 2% for imatinib), suggesting that imatinib is better in patients with severe cardiovascular diseases [30] (Figure 1).

### 3.4. Tracing TKI response in CML

After TKI treatment in the first phase for CML patients, certain evaluations are required. The tests needed for these evaluations are as follows: full blood count and peripheral blood smear (to evaluate hematological response), physical examination, cytogenetic analysis (to evaluate cytogenetic response), and quantitative Bcl-Abl1 analysis (to evaluate molecular response).

The cytogenetic assessment needs to be started as soon as the patient is diagnosed and needs to be repeated at the 3rd and 6th months. Then the assessment needs to be done once every 6 months for as long as CCyR is not observed. Similarly, molecular monitoring needs to be continued once every 3 months as long as MMR is not verified. After verifying the response, the assessment needs to be repeated once every 3–6 months. This test needs to be done with RT-QPCR, which may be an approved and helpful assessment tool but has the possibility of demonstrating different findings in each laboratory or even in the same laboratory (Table 2).

**Table 2:** Response evaluation with first-line therapy (ELN 2013).

	Optimal response	Warning	Failure
Baseline	--	High Sokal risk/Hasford score	
3 months	BCR-ABLIS ≤10% Ph+ ≤35% (PCyR)	BCR-ABLIS >10% Ph+ 36-95%	No CHR Ph+ >95%
6 months	BCR-ABLIS <1% Ph+ 0% (CCyR)	BCR-ABLIS 1-10% Ph+ 1-35%	BCR-ABLIS >10% Ph+ >35%
12 months	BCR-ABLIS ≤0.1% (MMR)	BCR-ABLIS 0.1%–1%*	BCR-ABLIS >1%* Ph+ >0%
Any time	MMR or better	CCA/Ph- (-7 or 7q-)	Loss of CHR Loss of CCyR Loss of MMR Confirmed loss of MMRMutations CCA/Ph+

BCR-ABL1 levels in patients under TKI treatments have been observed as determinative parameters, where BCR-ABL1 levels above 10% at the 3rd month are a negative determinative factor for the patient. There have been studies demonstrating this with various TKIs, such as GIMEMA and German CML IV (imatinib), DASISION (dasatinib), and ENESTnd (nilotinib) [10,11,31,32]. It was also stressed in the ELN 2013 recommendations that patients with BCR-ABL1 levels above 10% after the 3rd month are at critical risk and close monitoring is needed until the 6th month. For patients showing no complete hematological response and/or cytogenetic response, switching to another inhibitor might prove helpful, where imatinib should be dropped for one of the second-generation TKIs or its dose should be increased. If the patient was already being given one of the second-generation TKIs, switching to another one might prove helpful [18]. The recommendations state that better and quicker response rates have been seen in changing to the second-generation TKIs, though long-term survival benefits are still to be reported.

The recommendations further suggest that observing BCR-ABL1 levels under 1% after the 6th month or under 0.1% after the 12th month shows the ideal response desired, whereas if BCR-ABL1 levels stay above 10% and/or Ph chromosome is observed to be above 35% at the 6th month it means a failure in treatment, requiring a modification in the treatment method.

Dasatinib and nilotinib have been observed to be better at preventing the progression of CML. The key factors in the treatment of the disease are efficiently managing both the side effects of the inhibitors and the increased costs [7,8].

## 4. Second-line TKI treatment for CML

With all the benefits of TKIs, one problem with using them in treatment is, as mentioned before in this paper, patients developing a resistance to the drugs. Other than resistance, intolerance, less-than-optimal response, and relapse following first response are also existing problems. If a patient develops a resistance to imatinib, the treatment can be continued with one of the second-generation TKIs thanks to the lack of cross-intolerance.

As stated above, patients with BCR-ABL1 levels above 10% or Ph levels above 65% at the 3rd month are in a warning stage where replacing imatinib with one of the second-generation TKIs may prove useful, even though there is no information regarding long-term survival effect. For patients with BCR-ABL1 levels below 10% at the 3rd month or below 1% at the 12th month, continuance of the treatment is up to the clinician based on adherence problems, the rate of BCR-ABL1 decline, and the closeness to the critical levels of BCR-ABL1 depending on the time since the treatment started. Failure to achieve the aforementioned levels of BCR-ABL1 within the first 3 months puts patients in a risky position regarding progression of disease, but they can still keep using the same dose of the inhibitor for the next 3 months. For these patients, the necessary considerations in the following period would be mutational examination, allogeneic HCT assessment, and bone marrow cytogenetics evaluation (3rd month for MCyR/12th month for CCyR).

Patients with BCR-ABL1 levels above 10% at and after the 6th month and above 1% at the 15th month are considered resistant to the inhibitors. For these patients, HCT assessment is suggested (at the discretion of a transplant specialist, with the possibility of an HLA test being required) and alternative treatment methods need to be considered, which will be discussed further in the following sections. 

## 5. Third-line treatment for CML

With the failure of second-line treatment as in the mentioned response rates given above, proceeding to third-generation TKIs and/or allogeneic stem cell transplantation (allo-SCT) is authorized. In order to detect the occurrence of a BCR-ABL1 domain mutation in CML cells and whether the patients have moved on to the third phase (blastic phase), bone marrow biopsy needs to be done. Evaluating the second-generation TKIs and bosutinib and ponatinib in addition to them, along with the observed mutation, should demonstrate which drug would be useful in the following phase.

With a toxicity profile unlike any other inhibitor, bosutinib is a possible choice for patients not responding to dasatinib and nilotinib. Limiting its use is only one side effect, which is diarrhea, leading to myelosuppression and hepatotoxicity [33].

Bosutinib is not effective in T315I mutations, as with the second-generation TKIs, where the only remaining option will be ponatinib. If the desired response rates are still not observed after the induction of ponatinib, allo-SCT needs to be performed in the event that a donor is present. Ponatinib has very low side effect occurrence at 15–30 mg/day, with vaso-occlusive disease, skin rashes, and thromboembolism being seen in rare occasions [34].

## 6. Withdrawal of TKIs in CML

Among other studies on the effects of stopping TKI treatment, EURO_SKI stands out for having the largest population, where 821 CML patients who were treated with imatinib, dasatinib, or nilotinib as first-line treatment had the drugs discontinued and the survival rate without relapse was 52% at 2 years after discontinuance. Following the withdrawal of the drugs, 86% of the patients in the study stopped showing MMR, and 81% of the patients started showing response again after starting reuse of the drug. The study proposed that stopping TKIs would be safe as long as close monitoring is available [35].

In another study (STIM), patients using imatinib for 2 years and showing MMR had the medication discontinued and 61% of them showed deterioration at the 6th month following withdrawal. The same percentage of patients started showing response following the restarting of the drug, with decreases in BCR-ABL1 levels being observed in some others [36]. In the TWISTER study, 42% of 40 patients showed survival without any deterioration following the 2 years after withdrawal. In that study, every patient demonstrated response after the drug was restarted, showing that the alleviations and deteriorations observed were changing in each study [37].

Another study (ENEST) discontinued nilotinib in patients following a year of response, where 51.6% of them kept showing response for 2 more years, and resuming the drug showed responses in the majority of the rest [38].

For young or pregnant patients or those for whom treatment needs to be stopped after a period of time, using the second-generation inhibitors as frontline treatment would prove better, as we know so far that these drugs demonstrate longer response times following withdrawal [39].

Molecular cure levels are quite low in patients receiving inhibitor treatments. Various studies try to achieve CML stem cell control or resistant leukemic cell disposal. In patients with lower and medium levels of CML risk, imatinib and dasatinib are the two drugs that lead to better MMR along with Peg-IFN [40,41]. There is also information regarding increased apoptosis rates along with venetoclax and other some inhibitors in patients receiving imatinib, although wider population studies need to be conducted to obtain proof for clinical use [42].

## 7. Managing the blastic phase in CML

**Figure 2:**
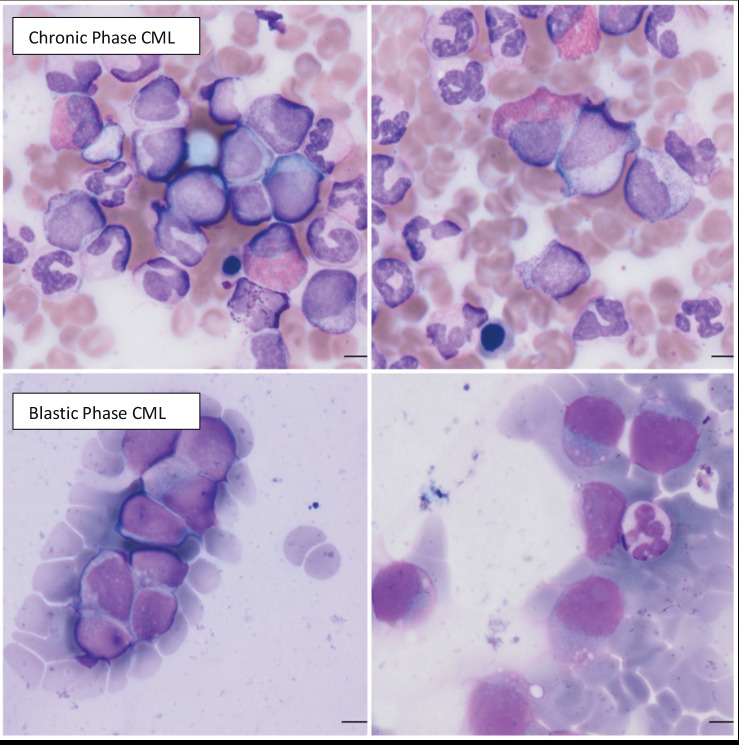
Histopathological demonstration of the myeloid expansion in chronic phase CML (upper panels), resulting in the advanced blastic crisis of CML (lower panels) with failure of tyrosine kinase inhibitors (courtesy of Professor Ayşegül Üner, MD, from Hacettepe University Medical School, Ankara, Turkey).

Prior to the TKI era, the blastic phase was unavoidable for CML patients, even though the inhibitors somehow led to increases in the time duration of the first phase. Today, occurrence of the blastic phase is about 1% annually, for which the suggested treatment objective is to either push the disease progression back into the chronic phase or to provide alleviation for the patient (Figure 2).

 In patients progressing on to the second or third stage, suggested methods include the use of dasatinib or ponatinib, which can be coupled with chemotherapy to increase the chances of survival and response. Considering the blastic phase in two types, as myeloid and lymphoid, suggested treatments are anthracyclines and cytosine arabinoside for the former and vincristine and prednisolone for the latter. The only known curing treatment method is allo-SCT (10% to 40% cure rate), if a donor is available, and further TKI use afterwards has showed decreased deterioration and DLI need. A study conducted with 477 CML patients in the third phase demonstrated that coupling TKI treatment, chemotherapy, and allo-SCT yielded the most successful results by 46%. In patients who start directly in the second phase (accelerated phase) and received TKIs, survival rates are between 60% and 80%, higher than the rates of patients progressing on to this stage following the chronic phase.

For patients who do not respond to the inhibitors and worsen throughout the application of ideal treatment methods, close monitoring is suggested, as these patients are to be considered highly risky for progression on to the third stage, knowing that there is yet to be an ideal method in managing this phase.

## 8. Future aspects and suggestions for CML

The subjects of new studies on CML patients include the effectiveness of methods like gene expression profiling, next-gen genomics, genetic polymorphisms, multidrug resistance genes, and existing BCR-ABL kinase domain mutations [43]. Future studies should aim to ensure reliable MMR rates and enable continuity for patients without the need for keeping up treatment. Apart from the tests, regarding coupling the TKIs with certain other agents (cl-2 inhibitors, protein synthesis inhibitors, or Peg-IFN) that were referred to multiple times in this paper, other combinations of the existing methods of treatments, or possibly discoveries of new agents, may ultimately aid in finding a cure for CML.
